# Supplementary material for: Scaling of Memories and Crossover in Glassy Magnets
Source: Sci Rep. 2017 Sep 21;7:12053. doi: 10.1038/s41598-017-12187-9 (PMC5608711; doi:10.1038/s41598-017-12187-9)

**Supplementary Information**

**Scaling of Memories and Crossover in Glassy Magnets**

A. M. Samarakoon, M. Takahashi, D. Zhang, J. Yang, N. Katayama, R. Sinclair, H. D. Zhou, S. O. Diallo, G. Ehler, D. A. Tennant, S. Wakimoto, K. Yamada, G-W. Chern, T. J. Sato, S.-H. Lee

**This PDF file includes:**

1. Experimental procedure of the Thermo-Remanent Magnetization (TRM)
2. Neutron Scattering Methods

Figures S1-S3

1. **Experimental procedure of the Thermo-Remanent Magnetization (TRM)**

The Figure S1 shows the DC susceptibility data obtained from Fe_1.02_Se_0.15_Te_0.85_ and La_1.96_Sr_0.04_CuO_4_ that exhibit their glassy transitions at low temperatures.

The Thermo-Remanent Magnetization (TRM) data, shown in Figure 1 and Figure 3 of the main text, and Figures S2 and S3 of the Supplementary Information, were collected using the following procedure. First, each sample was cooled down from well above the freezing temperature, $T_{f}$, to base temperature with a single stop for a period of time, $t_{w}$, at an intermediate temperature $T_{w}$ below $T_{f}$ under zero field. Once cooled down to base temperature, the thermo-remanent magnetization is measured by applying a small field of a few gauss upon heating at a constant rate. For all the measurements reported in this paper, we used a Superconducting Quantum Inference Device (SQUID) magnetometer, Quantum Design MPMS-XL5 equipped with the ultra-low-field option together with the environmental magnetic shield. Since it is necessary to have zero-field conditions at the sample during the cooling process including the waiting at an intermediate temperature, the remanent magnetic field at the sample position was measured by the instrument’s fluxgate, and has been eliminated by introducing a compensating field using non-superconducting DC coil to get the remaining uncompensated magnetic field less than 0.005 G at the sample position. After that, a small DC magnetic field of 3 G was generated by the DC non-superconducting coil and applied to the sample during the TRM measurements.

Figure S3 shows that for the spin jam systems the memory effect with $t_{w} = 10$ hrs is maximal when the waiting temperature $T_{w} \sim0.7 T_{f}$ and it becomes weaker for other values of $T_{w}$ over a wide range of $T_{w}$.

1. **Neutron Scattering Methods**

For the neutron scattering study of *Cu*Mn2%, the Backscattering Spectrometer (BASIS) at Spallation Neutron Source (SNS) was used. A 10 g polycrystalline sample of *Cu*Mn2% was sealed in a standard aluminum (Al) and was cooled in a standard liquid He-4 cryostat. During the measurements, the wavelength of scattered neutrons was fixed to be $6.2 Å$ by silicon analyzer crystals, yielding an elastic energy resolution of $\sim4 \mu eV$. For the neutron scattering study of Na_2_Ir_0.89_Ti_0.11_O_3_, the Cold Neutron Chopper Spectrometer (CNCS) at SNS. A 2.3 g polycrystalline sample of Na_2_Ir_0.89_Ti_0.11_O_3_ was sealed in an Al annular can with thickness of 1 mm to reduce the neutron absorption by Ir, and was placed inside a standard liquid He-4 cryostat that can go down to 1.4 K. The wavelength of incident neutrons was fixed to be $\lambda=5 Å$, yielding an elastic energy resolution of $\sim70 \mu eV$. Elastic magnetic Neutron scattering intensity $I_{elas}\left( Q,T \right)= \int_{-\omega_{0}}^{\omega_{0}} I(\omega,Q,T)d\omega$, where $\omega_{0}$ is the instrument’s elastic energy resolution has been determined by subtracting measurements done well above the freezing temperature$T_{f}$.

**Figure Captions**

**Figure S1:** High-Temperature bulk susceptibility (black) and inverse susceptibility (red) respectively, obtained from (a) Fe_1.02_Se_0.15_Te_0.85_ and (b) La_1.96_Sr_0.04_CuO_4_. The data above 120 K of Fe_1.02_Se_0.15_Te_0.85_ has been fitted to the Curie-Weiss law (red dash line) and the estimated Curie-Weiss temperature is -265.5(8) K. The measurements have done under magnetic fields of 0.01 T and 0.1 T respectively.

**Figure S2:** Bulk susceptibility, $\chi_{DC}= M/H$, where $M$ and $H$ are magnetization and applied magnetic field strength, obtained from (a) *Cu*Mn2% and (b) SrCr_9p_Ga_12-9p_O_19_ (p=0.97) with $H=3 Oe$. The $t_{w}=$1.5(5) min data is new while all other data for $t_{w}\geq6$ min are taken from Ref. 35.

**Figure S3.** Temperature Dependence of memory effect. $\chi_{DC}$ and $\left( M_{ref}-M \right)/{M_{ref}}$ measured for (a) Fe_1.02_Se_0.15_Te_0.85_ (b) La_1.96_Sr_0.04_CuO_4_, (c) Li_2_RhO_3_ (d) Na_2_Ir_0.89_Ti_0.11_O_3_ and (e) Y_2_Mo_2_O_7_, with $t_{w}=10 hrs$, at various waiting temperatures.

Fig. S1


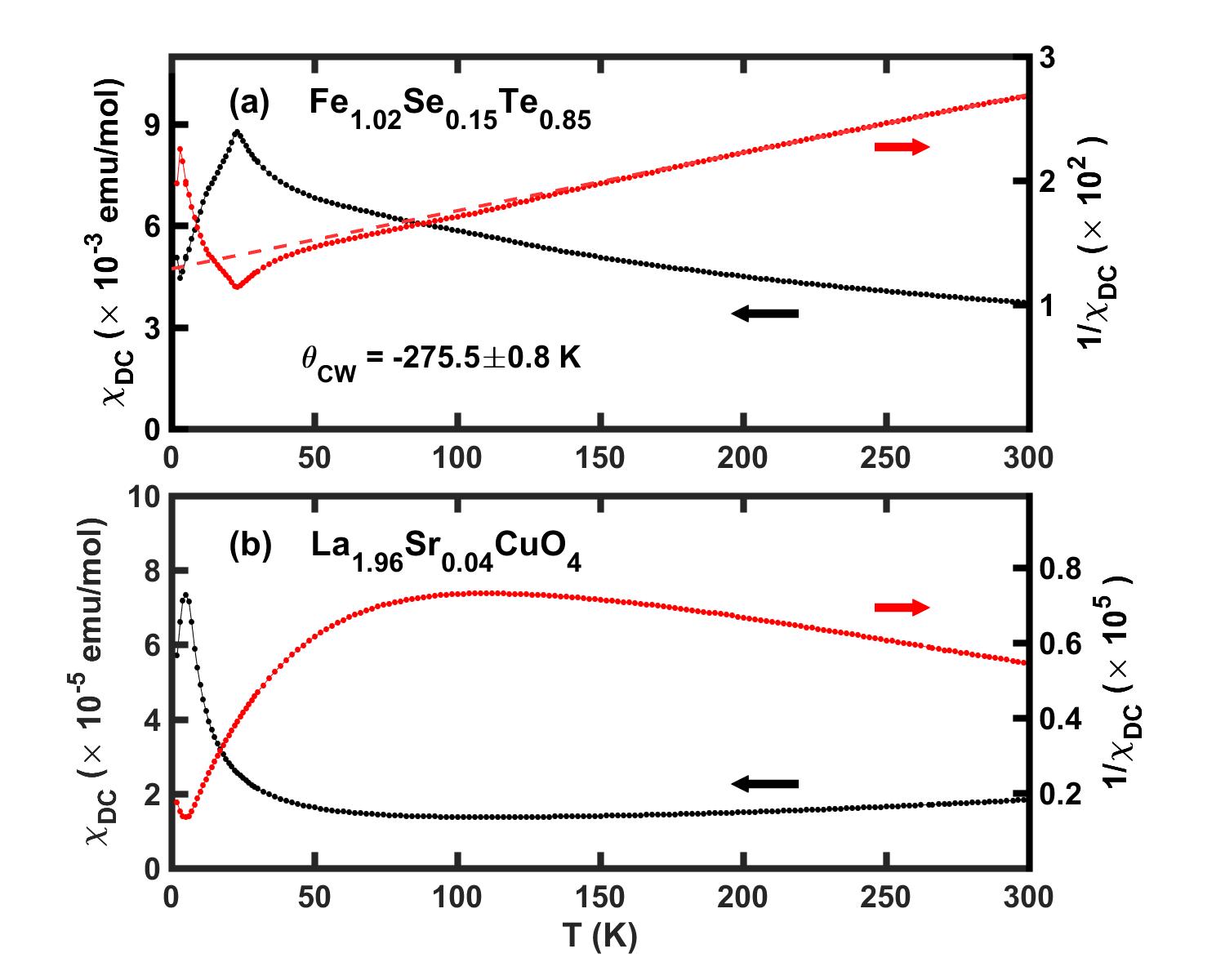


Fig. S2


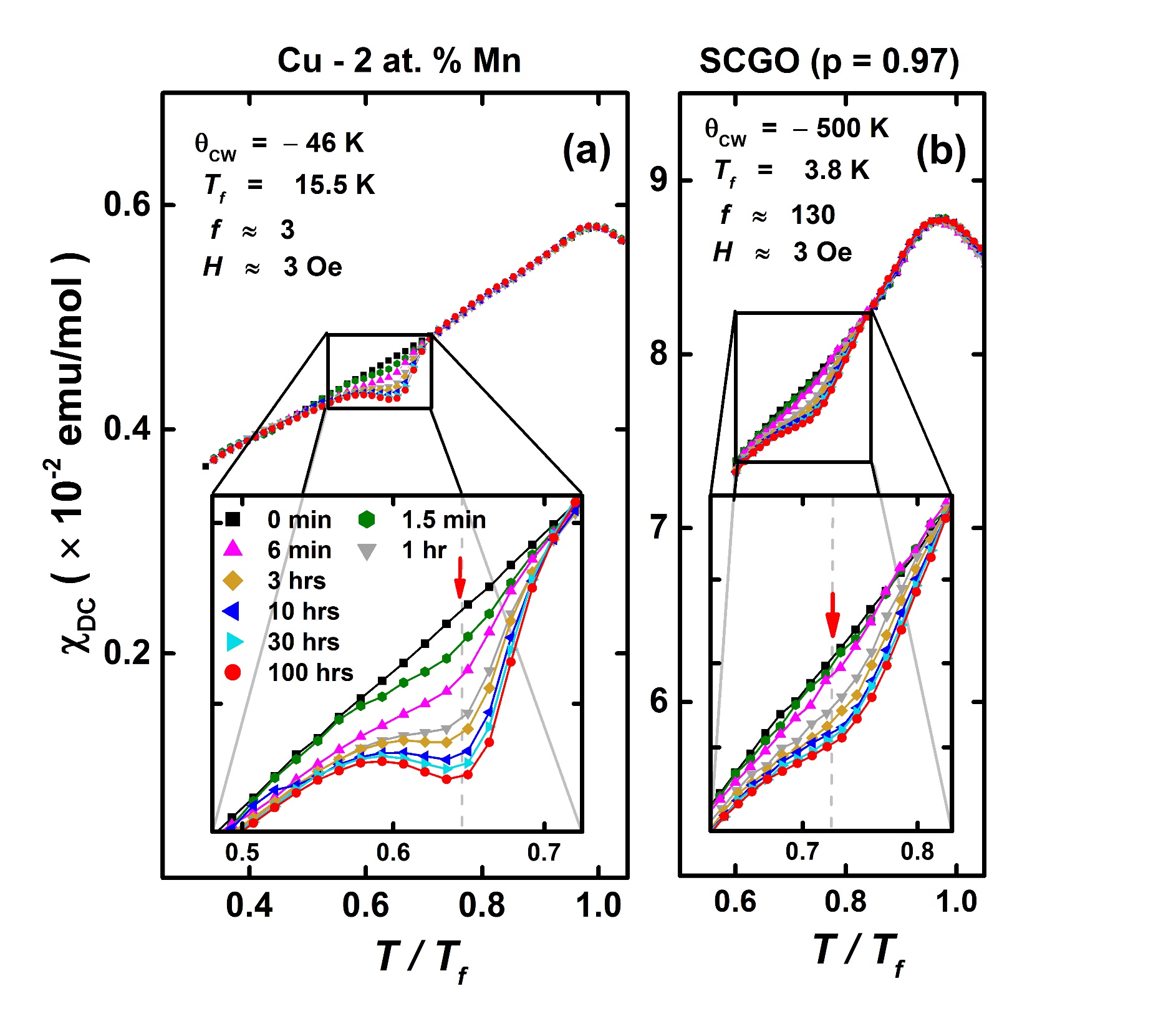


Fig. S3


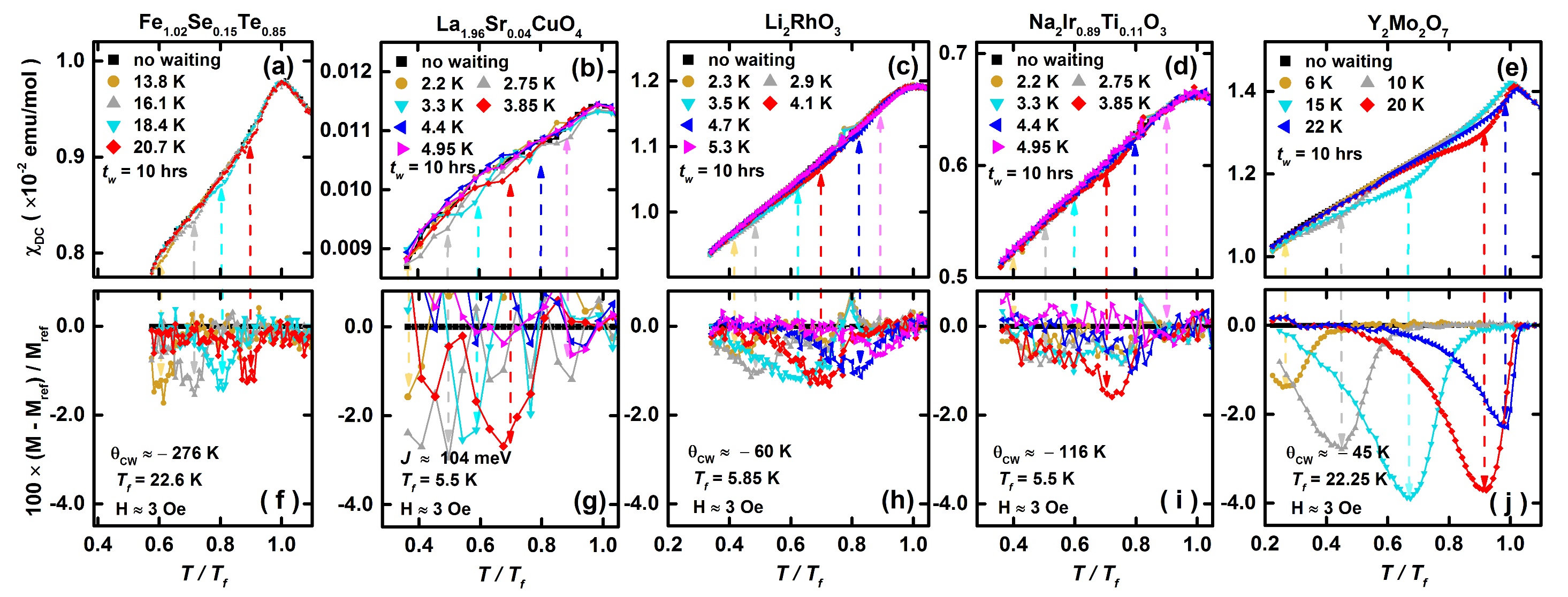

Supplement: Supplementary file 1 — Supplementary Information [file 41598_2017_12187_MOESM1_ESM.docx]
